# BMP-9 expression in human traumatic heterotopic ossification: a case report

**DOI:** 10.1186/2044-5040-3-29

**Published:** 2013-12-16

**Authors:** Guillaume Grenier, Élisabeth Leblanc, Nathalie Faucheux, Dominique Lauzier, Peter Kloen, Reggie C Hamdy

**Affiliations:** 1 Étienne-Le Bel Clinical Research Centre, Sherbrooke, QC, Canada; 2 Department of Orthopedic Surgery, Faculty of Medicine, Université de Sherbrooke, Sherbrooke, QC, Canada; 3 Shriners Hospital for Children, 1529 Cedar Avenue, Montreal, QC H3G 1A6, Canada; 4 Department of Surgery, Orthopedic Surgery Division, McGill University, Montreal, QC, Canada; 5 Department of Chemical and Biotechnological Engineering, Faculty of Engineering, Université de Sherbrooke, Sherbrooke, QC, Canada; 6 Department of Orthopedic Surgery, Academic Medical Center, Amsterdam, Netherlands

**Keywords:** BMP-9, Traumatic heterotopic ossification, Muscle resident stromal cells

## Abstract

**Background:**

Heterotopic ossification (HO) is defined as the abnormal formation of mature bone in soft tissue, notably skeletal muscle. The morbidity of HO in polytraumatized patients impacts the functional outcome, impairs rehabilitation, and increases costs due to subsequent surgical interventions.

**Case presentation:**

We present the case of a 34-year-old African male who developed severe HO around his right hip 11 days after a major trauma. Immunohistochemical analyses of resected tissue revealed that several BMPs were expressed in the HO, including highly osteogenic BMP-9.

**Conclusions:**

To the best of our knowledge, this is the first report of local BMP expression, notably BMP-9, in traumatic HO, and suggests that BMP-9, possibly through mrSCs, can contribute to HO formation in soft tissues when a suitable microenvironment is present.

## Background

Heterotopic ossification (HO) involves ectopic bone formation in soft tissues such as muscles and is often associated with trauma [1]. While the etiology of HO has been classified as neurogenic, traumatic, and hereditary, the exact pathophysiology of traumatic HO remains unknown. However, several critical factors such as progenitor cell populations, inductive factors, and a permissive environment may contribute to HO [2,3].

BMPs play a critical role in the osteoblastic commitment of mesenchymal cells and the induction of osteoblastic activity [4,5], and are potential candidates as inductive HO factors. BMPs are members of the transforming growth factor-beta (TGF-β) family [6]. More than 20 BMPs have been described to date, but experimental evidence indicates that only BMP-2, -6, -7, and -9 can induce osteogenesis, BMP-9 being one of the most potent osteogenic BMPs [7,8]. In addition, we recently reported that BMP-9 only induces HO in damaged muscle in a murine model [9]. Despite the fact that BMP-9 transcripts are barely detectable in human skeletal muscle [10], no studies have examined the expression of BMPs, including BMP-9, in human HO.

The BMP pathway is regulated by a negative feedback mechanism involving extracellular inhibitors such as noggin, chordin, and gremlin, membrane pseudo-receptors such as BAMBI, and the intracellular inhibitors Smad-6 and Smad-7 [11,12]. Based on studies by our group and others on the role of BMPs in normal and pathologic bone healing [13-17], we hypothesized that the balance between BMPs and their inhibitors may play a key role in the development of traumatic human HO.

In the present report, we describe the case of a patient with severe lower extremity trauma who developed HO. We determined the locations of BMPs, extracellular BMP antagonists, and BMP receptor BMPR1a by immunohistochemical staining. We propose a mechanism to explain the pathogenesis of trauma-associated HO and provide a novel perspective on the involvement of members of the BMP family.

## Case presentation

A 34 year-old African male was brought to the emergency department after jumping from the seventh floor of an apartment building during a police raid. Physical and radiological examinations revealed a C1 fracture, a carpal dislocation, and a T-type fracture of the right acetabulum.

On the day of admission (Day 0), an open reduction and internal fixation (ORIF) of the acetabular fracture was performed using a standard Kocher-Langenbeck approach associated with a trochanteric flip [18].

A revision for suboptimal reduction was performed on Day 10, followed by a second stage ORIF the next day using an ilioinguinal approach [19]. During the revision surgery, a mass of heterotopic bone was resected and sent for analysis. The patient did not receive NSAIDs or radiotherapy preoperatively. Clinical and radiological evaluations were performed for three and a half months postoperatively.

## Methods

### Tissue specimen

The tissue specimen harvested during the second surgery (Day 11) was processed for immunohistochemical and histological analyses.

### Histology and immunohistochemistry

The sample was fixed in buffered formalin overnight, decalcified in 10% EDTA (pH 7.2) for three weeks, embedded in paraffin, and sectioned using a Leica RM 2255 microtome (Leica Microsystems, Richmond Hill, ON, Canada). Following deparaffinization and hydration, the tissue sections were stained with Goldner’s trichrome to visualize nuclei (blue-gray), cartilage (red-purple), osteoid (orange-red), and mineralized bone (green).

The immunohistochemistry was performed as described previously [13-15]. Commercially available goat and rabbit pAbs and a mouse mAb (1:100 in 1% goat or 1% horse serum) were used to detect BMP-2, BMP-7, BMP-9, noggin, gremlin, BMPR1a, and BAMBI (Santa Cruz Biotechnology Inc., Santa Cruz, CA, USA). A list of the antibodies is given in Table 1.

**Table 1:** Primary and secondary antibodies used for the immunohistochemical staining procedure

**Primary antibody**	**Catalog number**	**Type**
BMP-2	sc-6895	Goat pAb
BMP-7	sc-34766	Rabbit pAb
BMP-9	sc-130703	Rabbit pAb
Chordin	sc-28964	Rabbit pAb
Gremlin	sc-28873	Rabbit pAb
Noggin	sc-25656	Rabbit pAb
BAMBI	sc-100681	Mouse mAb
BMPR1a	sc-5676	Goat pAb
**Secondary antibody**		
Biotinylated horse anti-goat IgG	BA-9500	
Biotinylated goat anti-rabbit IgG	BA-1000	
Biotinylated goat anti-mouse IgG	BA-9200	

### Grading of the immunostained sections

Immunohistochemical staining of cellular and extracellular proteins has been used in mandibular [20], long bone distraction osteogenesis [21-24], and bone healing [13-15] studies. We employed this technique to semi-quantitatively evaluate our results based on the percentage of positively stained cells using the following grading scheme: +, 25% of the cells stained positively for the protein of interest; ++, 25 to 50% of the cells stained positively; +++, 50 to 75% of the cells stained positively; ++++, more than 75% of the cells stained positively; -, no cells stained positively.

## Results

### Radiological and clinical follow-up

**Figure 1:**
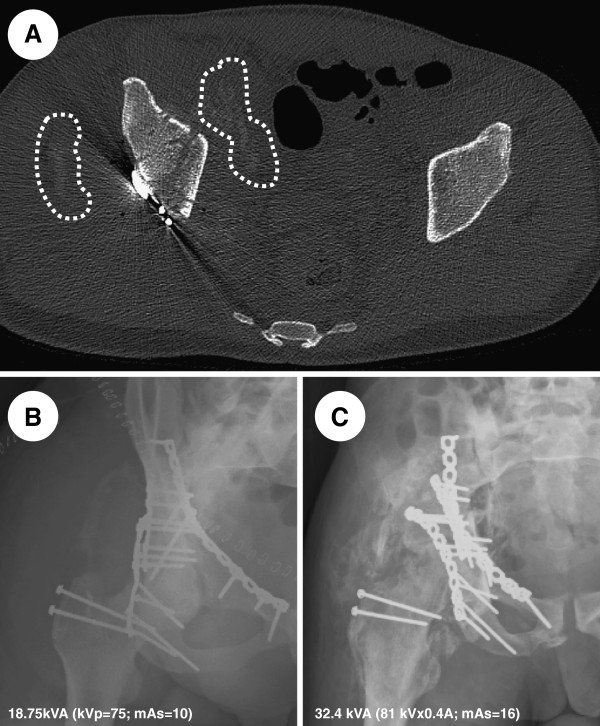
**Early and late radiological evidence of heterotopic ossification around the right hip. (A)** Axial computerized tomography (CT) scan cut of the acetabular region 11 days post-trauma. Note the denser soft tissues (encircled) surrounding the right fractured acetabulum. Radiographic AP view of the right hip at 14 days; **(B)** and three and a half months; **(C)** post-trauma. Note the progression of calcified soft tissues medially and laterally within the abductor muscles.

Computerized tomography (CT) imaging prior to the second surgery (Day 11) revealed the presence of dense ectopic tissue in the musculature (Figure 1A). A post-ORIF radiological examination (Day 14) revealed adequate reduction/fixation as well as the presence of dense tissue in the abductor muscles (Figure 1B). Three and a half months later, a repeat antero-posterior (AP) radiograph of the pelvis revealed a healed fracture and a Brooker radiologic stage III-IV HO (Figure 1C) [25]. While follow-up with this patient was limited, clinical notes indicated that he had a stiff hip gait.

### Macroscopic and microscopic tissue organization

**Figure 2:**
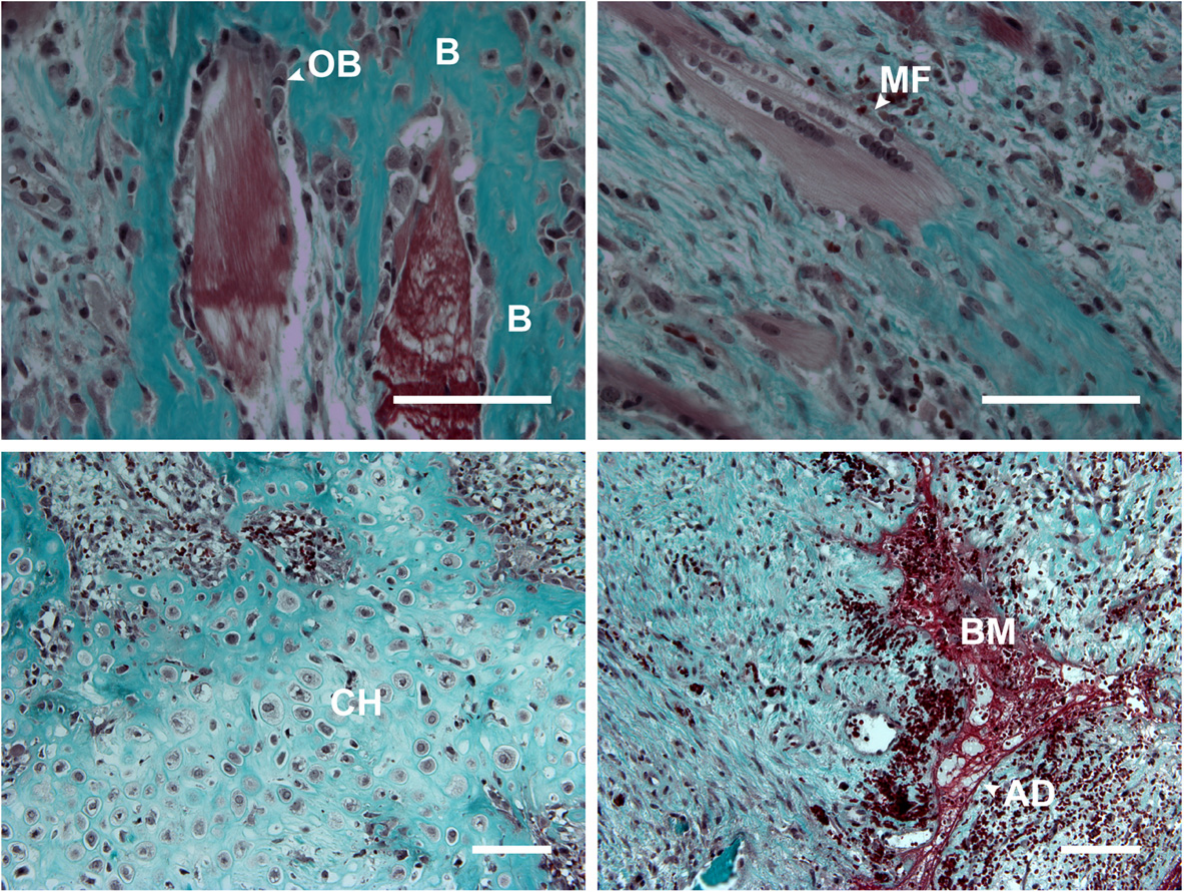
**Resected heterotopic ossification (HO) tissue with endochondral ossification.** Micrograph of a section of resected tissue stained with Goldner’s trichrome. AD, adipocyte; B, bone; BM, bone marrow; CH, chondrocyte; MF, myofiber; OB, osteoblast. Scale bar = 100 μm.

The resected tissue had a macroscopic rubbery texture on the day of the revision surgery (Day 11). A histological examination showed that the tissue was composed mainly of mature bone and cartilage (Figure 2). Adipocytes organized in a bone marrow-like structure in the ectopic bone were also observed. The structure was well vascularized, as previously reported [9,26]. We also observed muscle fiber remnants intertwined with the tissue.

### Expression of BMPs, BMP receptors and their antagonists

**Figure 3:**
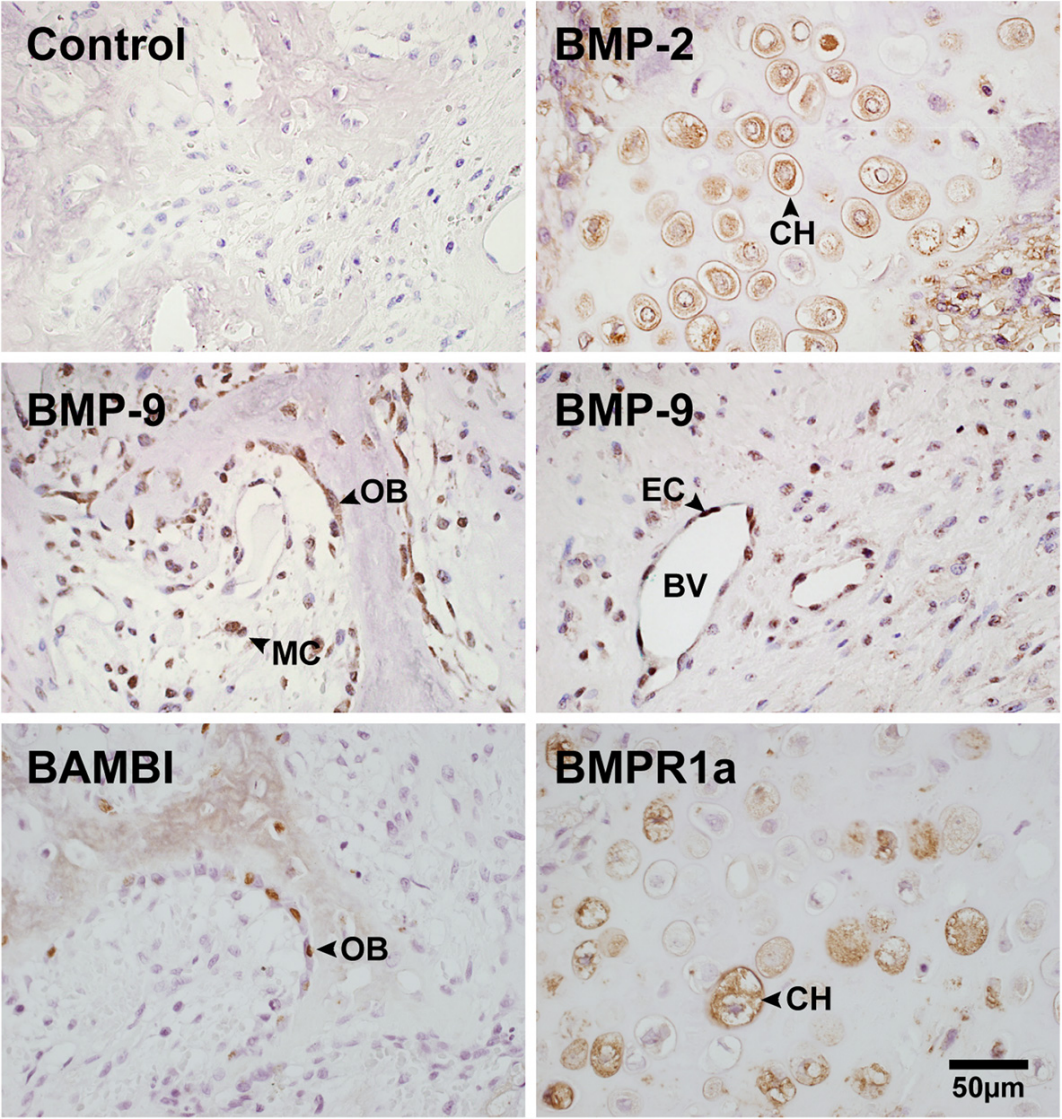
**Immunostaining of resected heterotopic ossification (HO) tissue.** Micrographs of resected tissue immunostained for various proteins in the BMP pathway. The control shows background staining. BV, blood vessel; CH, chondrocyte; EC, endothelial cell. MC, mesenchymal cell; OB, osteoblast.

Chondrocytes stained fairly strongly for BMP-2 (Figure 3). Interestingly, while BMP-9 was expressed in various cell types, the most intense staining was observed in osteoblasts followed by endothelial cells (Figure 3). The receptor antagonist BAMBI was mainly expressed in osteoblasts while BMPR1a was mainly expressed in chondrocytes. Noggin, chordin, and gremlin, the extracellular antagonists of BMP-2 and BMP-7, were mainly observed in chondrocytes and mesenchymal cells. See Table 2 for a summary of the immunostaining results.

**Table 2:** Immunolocalization of BMP receptors, agonists, and antagonists

**Antibody**	**OB**	**CH**	**FB**	**MC**
BMP-2	*+*	*++++*	*-*	*++*
BMP-7	*-*	*+*	*-*	*+++*
BMP-9	*++*	*+*	*-*	*+*
BMPR1a	*-*	*++++*	*-*	*-*
Chordin	*-*	*++*	*+*	*++++*
Gremlin	*-*	*++++*	*+*	*+++*
Noggin	*-*	*++++*	*+*	*++*
BAMBI	*++*	*-*	*-*	*-*

CH: chondrocytes; FB: fibroblasts; MC: mesenchymal cells; OB: osteoblasts.

## Discussion

**Figure 4:**
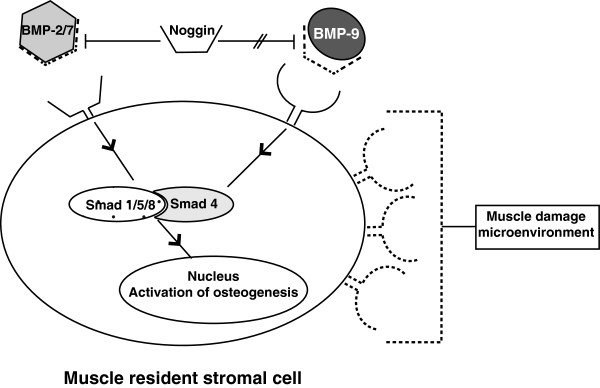
Diagram illustrating that BMP-9 may play a pivotal role in heterotopic ossification (HO) because of the more permissive environment of damaged muscle and the inability of BMP inhibitors in the tissue to alter the osteogenic program of multipotent progenitor cells.

In the present case report, we describe for the first time the expression patterns of several BMPs as well as BMP receptors and their antagonists in traumatic HO. We found that BMP expression patterns were cell type-dependent, with chondrocytes expressing the highest levels of BMP-2, mesenchymal cells the highest levels of BMP-7, and osteoblasts and endothelial cells the highest levels of BMP-9. This is the first time that BMP-9 has been shown to be expressed in human ectopic bone. This is an important finding since it has been previously reported that BMP-9 is the most osteogenic BMP both *in vitro* and *in vivo*[7,8]. Unlike BMP-2, which can induce HO in undamaged skeletal muscle, BMP-9 induces HO only in damaged muscle [9]. While BMP-2 and BMP-9 share the same pathway, they do not appear to be functionally equivalent in terms of inducing heterotopic bone in skeletal muscle. Two main factors seem to be responsible for this difference and make the involvement of BMP-9 in HO more plausible. The microenvironment of cells in damaged muscle is more permissive to the development of HO [9,27]. Since BMP-9 is involved in angiogenesis [28] and participates in endothelial cell proliferation, severe muscle trauma may promote higher local expression of BMP-9 and, as such, HO. In addition, despite the fact that BMP inhibitors were expressed in the sample we analyzed, osteogenesis still occurred. This might be because noggin, an effective antagonist of BMP-2 and BMP-7, cannot inhibit BMP-9 [29,30] (Figure 4).

The difference in BMP expression in healing fractures and in HO might also be explained by the nature of the cells involved. A number of studies using animal models [9,31] and human cells [32] have shown that muscle resident stromal cells (mrSCs) with high osteogenic potential contribute to HO. This finding has important clinical implications since mrSCs may be involved in fracture repair [33-35]. More importantly, non-unions are more likely to be associated with bones with minimal soft tissue coverage such as the tibia, and it has been proposed that non-unions could occur due to the lack of enveloping muscle tissue [36]. The present case report underlines the importance of concentrating future studies on identifying the true role of multipotent mrSCs and the pathway leading to their osteogenic commitment.

## Conclusions

We showed that BMPs are expressed in traumatic HO and that BMP-9 in particular may play a pivotal role in HO because of the more permissive environment of damaged muscle and the inability of BMP inhibitors in the microenvironment to alter the osteogenic program of multipotent progenitor cells. We propose that osteogenic multipotent progenitor cells, including mrSCs, and their signaling pathways should be investigated further with a view to developing prophylactic measures to prevent traumatic HO.

## Consent

Informed consent was obtained from the patient for the publication of this case report and any accompanying images.

## Abbreviations

AD: adipocyte; AP: antero-posterior; BMP: bone morphogenetic protein; BM: bone marrow; BV: blood vessel; CH: chondrocytes; CT: computerized tomography; EC: endothelial cell; FB: fibroblasts; HO: heterotopic ossification; MC: mesenchymal cells; MF: myofiber; mrSC: muscle resident stromal cell; NSAIDs: non-steroidal anti-inflammatory drugs; OB: osteoblasts; ORIF: open reduction and internal fixation; TGF-β: transforming growth factor-beta.

## Competing interests

The authors declare that they have no competing interests.

## Authors’ contributions

Collecting the data: DL and PK. Analyzing the data: GG, EL, NF, PK and RH. Interpreting the data*:* GG, EL, NF, PK and RH. Drafting the manuscript: GG, EL and NF. Approving the final version of the manuscript: GG, EL, DL, NF, PK and RH. GG, EL, DL, NF, PK, and RH are responsible for the integrity of the data analysis. All authors read and approved the final manuscript.
